# Measurement equivalence in mixed mode surveys

**DOI:** 10.3389/fpsyg.2015.00087

**Published:** 2015-02-05

**Authors:** Joop J. Hox, Edith D. De Leeuw, Eva A. O. Zijlmans

**Affiliations:** ^1^Department of Methodology and Statistics, Utrecht UniversityUtrecht, Netherlands; ^2^Department of Methodology and Statistics, Tilburg UniversityTilburg, Netherlands

**Keywords:** mixed mode survey, measurement equivalence, measurement invariance, mode effect, selection bias, propensity score adjustment

## Abstract

Surveys increasingly use mixed mode data collection (e.g., combining face-to-face and web) because this controls costs and helps to maintain good response rates. However, a combination of different survey modes in one study, be it cross-sectional or longitudinal, can lead to different kinds of measurement errors. For example, respondents in a face-to-face survey or a web survey may interpret the same question differently, and might give a different answer, just because of the way the question is presented. This effect of survey mode on the question-answer process is called measurement mode effect. This study develops methodological and statistical tools to identify the existence and size of mode effects in a mixed mode survey. In addition, it assesses the size and importance of mode effects in measurement instruments using a specific mixed mode panel survey (Netherlands Kinship Panel Study). Most measurement instruments in the NKPS are multi-item scales, therefore confirmatory factor analysis (CFA) will be used as the main analysis tool, using propensity score methods to correct for selection effects. The results show that the NKPS scales by and large have measurement equivalence, but in most cases only partial measurement equivalence. Controlling for respondent differences on demographic variables, and on scale scores from the previous uni-mode measurement occasion, tends to improve measurement equivalence, but not for all scales. The discussion ends with a review of the implications of our results for analyses employing these scales.

## Introduction

Mixed mode surveys, which combine different modes of data collection, such as, face-to-face, telephone, and web, are becoming standard data collection tools (Biemer and Lyberg, 2003, p. 208; De Leeuw, 2005; De Leeuw et al., 2008; Dillman et al., 2014, p. 13). Mixed mode survey designs are attractive, because they are cost effective and because they can be successful in reaching different kinds of respondents (De Leeuw, 2005; Blyth, 2008). As a result, they have the potential to decrease both coverage errors and nonresponse errors, thereby increasing the representativeness of the final (combined) sample at affordable costs (Couper, 2011).

However, a combination of different modes in one survey, be it cross-sectional or longitudinal, can lead to different kinds of measurement errors (De Leeuw and Hox, 2011). An important distinction is in errors caused by the design and implementation of the survey and in mode inherent errors (De Leeuw, 2005; Dillman and Christian, 2005; Roberts, 2007). The former can be prevented; for instance, in the design phase survey questions are sometimes constructed differently for each mode (e.g., offering do-not-know in one mode but not in another). As a result respondents in particular modes are presented with different question formats, which will produce differences in responses. To avoid these question-format mode effects, Dillman et al. (2014, chapter 11) advocate the uni(fied)-mode design where equivalent questionnaires are developed for each mode in a mixed mode study. Mode effects can and should be reduced in the design phase as far as possible (see also De Leeuw et al., 2008 on designing for mixed-mode studies).

Mode inherent errors are part of the mode itself (Berzelak, 2014) and are not avoidable by clever design. A clear example is the way questions are presented to the respondent; this can be done visually or aurally; furthermore when questions are presented visually, the visual lay-out may convey extra information (e.g., Christian et al., 2007). As a consequence respondents in an interview survey may interpret the same question differently from respondents in an online survey and give a different answer, just because of the mode used. Another example is the presence or absence of an interviewer and its influence on sensitive questions (Dillman et al., 2014, chapter 8; Tourangeau et al., 2000, chapter 10).

We distinguish two different types of mode inherent effects on measurement (De Leeuw, 1992, chapter 7; Jäckle et al., 2010). First there are mode effects that only shift the response distribution; this produces differences in the mean or variance of scale scores between survey modes, but does not change correlations. The second mode effect is a change in the question-answer process and as a consequence the question is interpreted and answered differently. This can be the result of avoidable mode differences in wording, but also of mode inherent differences between aural and visual presentation. The latter has the potential to produce measurements of constructs that are not equivalent between modes. In the worst case the instruments reflect qualitatively different constructs across modes. Both types of mode effect will be investigated in this study.

In addition to the measurement effect of survey mode on the response process, differential nonresponse across modes may play a role. Due to differential nonresponse, different types of respondents tend to end up in the different modes, even in randomized mode experiments. If these differences in sample composition across modes coexist with mode effects, this leads to confounding of substantive and methodological effects (Klausch et al., 2013; Vannieuwenhuyze and Loosveldt, 2013). For example, assume that we use a mixed mode web–interview survey to study drinking behavior. Web surveys attract younger respondents than traditional interviews (Couper, 2000; Mohorko et al., 2013). In addition, web surveys also elicit less socially desirable responses (e.g., Link and Mokdad, 2005). Since in our example the web mode is confounded with age, if the web respondents report more extreme drinking behavior we cannot distinguish whether the mixed mode data reveal a real relation between extreme drinking and age or if this is just the result of less socially desirable answers over the web.

Panel surveys pose their own challenge in this respect. Given the high costs of longitudinal panel surveys, there is a growing interest in applying mixed-mode data collection methods in such surveys (Dex and Gumy, 2011). Obviously, in longitudinal surveys that focus on measuring and explaining change, assessing and correcting mode effects is essential for a correct interpretation of trends over time. Compared to cross-sectional surveys, longitudinal surveys are in a special position. To assess selection effects, access is needed to auxiliary data not affected by mode effects. These data may come from elsewhere (e.g., a register), or the specific data are simply assumed to be unaffected by mode. Often the assumption is made that biographic information, such as, sex and age are measured without error. Even if that assumption is true, or if we have access to this information from a register, the problem remains that biographic variables usually are only weakly related to the substantive variables of interest and are therefore not very effective in assessing or correcting mode effects (Vannieuwenhuyze and Loosveldt, 2013). In longitudinal surveys that incorporate mixed mode data collection, preferably the first data collection occasion uses a single mode face-to-face interview, because this mode has the highest response rate compared to other or mixed modes (Hox and De Leeuw, 1994; Lozar Manfreda et al., 2008). The subsequent measurement occasions then shift to a less expensive mixed mode data collection. When this longitudinal survey design is followed, the first round of data collection provides a single mode data set that contains the substantive variables of interest measured with a constant mode effect. As a result, analysts have access to strong information to assess and correct mode effects.

Mode effects have been studied extensively for the traditional modes: face-to-face, telephone, and self-administered (e.g., mail) surveys not involving Internet. Most of these studies investigate simple mode effects, such as shifts in the response distributions of single questions, amount of missing data, or effects on sensitive questions. These studies typically find small differences, often indicating a dichotomy between survey modes with and without an interviewer (Groves, 1989; De Leeuw, 1992). When web surveys are added to the comparison, they tend to behave as self-administered paper-and-pen surveys. For an overview of such studies we refer to Christian et al. (2008), De Leeuw and Hox (2011), and Tourangeau et al. (2013, chapter 7).

Investigating measurement effects of data collection modes is difficult when individual questions are examined; repeated measures designs with several repeated measurement occasions are needed to distinguish between systematic and random measurement errors and true change over time. Alwin (2007) discusses the requirements for such designs, but also notes that their application to mode effect studies remains a challenge. When multi-item scales are involved, measurement equivalence can be investigated using models based on Item Response Theory (IRT) or Structural Equation Modeling (SEM). Since these models are closely related (Glockner-Rist and Hoijtink, 2003), we will only discuss measurement equivalence in mixed mode surveys using SEM. Given the potential confounding of selection effects by differential nonresponse in modes and by mode effects on measurement, we review only studies that also pay attention to differences in sample composition (i.e., selection effects) between the modes.

The question if measurement equivalence may be assumed, naturally occurs in cross-cultural comparisons across countries, where this is generally investigated in a Multigroup Confirmatory Factor Analysis (MCFA) using multigroup SEM. The assessment of measurement equivalence typically proceeds in steps (Jöreskog, 1971; Meredith, 1993; Vandenberg and Lance, 2000). The first step tests if the same factor model applies in different groups, traditionally countries, but in this particular study, modes are seen as groups. This is the weakest form of equivalence, configural equivalence, merely assuming that the different groups display the same pattern of factor loadings, i.e., the same number of factors, and these factors can be interpreted as similar because they have comparable loadings for their empirical indicators. The second step tests if (most of) these factor loadings can be constrained to be equal across all groups. If this holds we have (partial) *metric equivalence* (Vandenberg and Lance, 2000). When (partial) metric equivalence is achieved, one can validly test if the same structural model holds in all groups. The third step tests if (most of) the measurement intercepts can be constrained equal across all groups. If this holds we have (partial) *scalar equivalence* (Vandenberg and Lance, 2000). Full scalar equivalence is called strong measurement invariance in the psychometric literature (Meredith, 1993) and implies that the relationship between the observed score and the unobserved score on the latent factor of a person does not depend on group membership (Mellenbergh, 1989). Full scalar equivalence or strong measurement invariance allows variances and covariances between latent and observed scores to be different across groups. The psychometric literature also distinguishes strict measurement invariance, where the residual variances are also identical across groups (Millsap and Meredith, 2007). Since strict invariance is not necessary for valid comparisons across groups, we do not pursue strict invariance here.

When (partial) scalar equivalence is achieved, one can then investigate whether the latent means or actual sum scores differ across the groups (step 4). For a valid comparison of groups it is not necessary that error variances be constrained equal (strict measurement equivalence), but if this constraint holds, this has the advantage that we are measuring with equal precision across groups. Regarding the minimal requirements for partial invariance, both Byrne et al. (1989) and Steenkamp and Baumgartner (1998) state that for each construct, in addition to the marker item that defines the scale –with marker item loading fixed at 1 and intercept fixed at 0–, at least one more indicator must have invariant loadings and intercepts across the groups. When the groups to be compared are different modes in a randomized mixed mode survey, the fourth step is extremely important. This fourth step tests if the latent mean or sum scores in different modes are equal. If not, we may have measurement equivalence, but the different modes still result in a response shift, with some modes reporting higher scores than other modes. This response shift points toward either a systematic bias in one of the modes or different systematic biases across modes.

What is known about measurement (in)equivalence across different modes? Probably the first mode experiment employing multigroup SEM is De Leeuw (1992, see also De Leeuw et al., 1996), who analyzed data from a national Dutch probability sample. They find non-equivalence, particularly between the mail survey mode on the one hand and the interviewer based face-to-face and telephone modes on the other. Although this study used random assignment to modes, there were small differences on age and gender, which were not controlled for in the SEM analyses.

Klausch et al. (2013) review empirical studies that evaluate measurement equivalence using MCFA following the sequence of steps outlined above. They report that comparisons of web and paper-and-pen surveys generally find full scalar equivalence and that measurement differences (i.e., nonequivalence) are more often found in comparisons of modes that do with modes that do not involve interviewers. However, most of the reviewed studies involve small samples of specific populations such as students or employees and not all of these studies control for potential selection effects. Below, we review in more detail studies that involve general populations and exert good control of selection effects.

Klausch et al. (2013) report a mode experiment in a crime victimization study using a random sample from the general population in The Netherlands. The respondents were randomly assigned to one of four modes: face-to-face, telephone, mail, and web; propensity scores based on eight socio-demographic variables were used to control for selection effects. The response categories formed either a three- or a five-point Likert scale. The data were analyzed with a MCFA specifying the variables as categorical and employing weighted least squares estimation. This approach involves estimation of thresholds for the observed variables, which allows an evaluation of the way respondents choose specific categories in the different modes. Klausch et al. (2013) report that interviewer-based surveys differ from self-administered surveys in measurement characteristics, with different systematic bias and different amounts of random error. The self-administered modes (i.e., mail and web) have lower category thresholds, indicating a greater tendency to agree to questions. Furthermore, the self-administered modes have lower error variances, which results in higher reliabilities for these modes.

Revilla (2013) compares data from two different large scale surveys in the Netherlands (the Dutch LISS internet panel and the Dutch contribution to the face-to-face ESS survey), both using large random samples from the general adult population. Using MCFA, she finds full scalar equivalence, including equal means on the latent variables, for four separate concepts. Although there is no explicit control for selection, Revilla (2013) reports that the two samples are very similar with respect to gender, age and education. Saris and Revilla (2013) analyze six Multi-Trait Multi-Method (MTMM) matrices from the same data sources. They focus on the *quality* of the responses, which they define as the strength of the relationship between the latent variable and the corresponding responses (Saris and Revilla, 2013, p. 2). They report finding few and small differences, if differences are found the questions in the LISS web survey have a higher quality than the corresponding questions in the face-to-face ESS survey.

Gordoni et al. (2012) investigate mode effects in a general survey of the Arab population in Israel, using face-to-face and telephone interviews. The survey topics concerned coexistence among the Arab minority in Israel, a topic that is potentially sensitive. For each survey mode an independent probability based sample was drawn. In addition, relevant demographic variables were included in the analysis as covariates. Gordoni et al. (2012) report full metric and partial scalar equivalence across the two modes. Measurement errors tended to be higher in the telephone mode than in the face-to-face mode.

Heerwegh and Loosveldt (2011) compare Likert scale responses in a national crime victimization study in Belgium. They use a mixed-mode design with telephone, mail and web modes. Assignment to modes was not random, but depended on the availability of a landline telephone number in the sampling frame. To control for differences between the modes, gender, age, education, job, and type of residence are included in the model as covariates. Conditional on these covariates, Heerwegh and Loosveldt (2011) report complete scalar equivalence between the combined mail/web and telephone modes. However, they do find a difference in the latent factor means: in the telephone mode the respondents show a more favorable attitude toward the police. Heerwegh and Loosveldt (2011) interpret these findings as the result of social desirability in the interviewer-based telephone survey.

Chang and Krosnick (2009) describe a national field experiment where the same questionnaire is administered to an RDD telephone sample, an Internet probability sample, and an Internet nonprobability, volunteer panel. After weighting all samples toward national demographics, they report that the two probability samples were more representative than the nonprobability sample, a difference that did not completely disappear after weighting. Compared to the probability based Internet sample, the telephone sample produced data that contained more random measurement error, more satisficing behavior, and more social desirability bias. These results were confirmed in a later laboratory study using students (Chang and Krosnick, 2010).

Summarizing: our review of large scale mode experiments that examine measurement equivalence across survey modes shows that all studies confirm configural measurement equivalence. This is not surprising, since all mode experiments investigated the measurement equivalence of well-established scales, scales with proven reliability and validity. It would in fact be rather shocking if any mode would completely alter the structure of a reliable and valid scale that has been established in previous research. Many of the studies reviewed report full or at least partial scalar equivalence. When partial equivalence is found, the problems are more often situated in the intercepts or with ordinal measurement with the thresholds, which indicates just a shift in the response distributions across modes, and not in the factor loadings. Several studies report that error variances tend to be larger in interviewer based modes, especially in telephone surveys. It can be argued that the higher reliability in self-administered and especially internet modes simply reflects a common method effect, because in web and mail surveys several questions are usually presented together on one screen/page, instead of sequentially as is the case in interviews. This can enhance the intercorrelations between questions and thus increase the reliability, without increasing the validity. However, the studies of Saris and Revilla (2013) and Chang and Krosnick (2010) both suggest that the validity also increases.

Finally, *all* studies that report on demographics find small but systematic differences between the modes, even in randomized experiments. This finding confirms the importance of controlling for sample differences between modes in all survey mode experiments. In our study, we use propensity scores with covariate adjustment, a method that is suitable for controlling a potentially large number of covariates simultaneously. Our application of propensity score adjustment is described in detail in the next section of this paper.

The study reported here addresses three related research questions. The data source is a large longitudinal survey, which in its third wave of data collection changed over from single mode face-to-face to a mixed mode data collection. The first research question is whether the scales used do show measurement equivalence. If measurement inequivalence is found, this can be the effect of selection or of measurement differences due to mode. The second research question therefore is to what extent measurement equivalence improves if selection on demographic variables is controlled, and the third research question is to what extent measurement equivalence improves if scale scores from the earlier single mode data collections are added to the control variables.

## Data and analysis methods

### Data

The data are from the Netherlands Kinship Panel Study (NKPS). The NKPS is a large-scale, nationally representative panel study on kinship in the Netherlands. Three waves of data collection have been conducted: wave 1 in 2002–2004, wave 2 in 2006–2007, and wave 3 in 2010–2011. Below we describe the data collection procedures briefly; full detail on design and fieldwork is available in the codebooks and questionnaires published on the NKPS homepage (www.nkps.nl), which also explains how researchers can obtain access to the NKPS data files. The NKPS data collection is funded by the Netherlands Organization for Scientific Research (NWO) and complies with standard NWO ethical requirements such as voluntary participation and informed consent.

The main NKPS wave 1 net sample consists of 8161 individuals who had responded to a face-to-face computer assisted interview (CAPI). Self-completion paper questionnaires were used to collect additional data from family members. In our analysis, we use only the data provided by the primary respondents, which are denoted as *anchor* in the NKPS files. In the second wave, a mixed mode design was introduced, where respondents were first approached for a face-to-face interview (CAPI), and computer assisted telephone interviewing (CATI) or computer assisted web interviewing (CAWI) were offered only at the end of the data collection period to sample members who had previously refused to participate or who had not been reached. This resulted in a net sample of 6091 individuals for the second wave. Very few respondents used the alternative options: about 3% used CATI and about 2% used CAWI. In our analysis, we have used only the face-to-face data from wave 2.

The NKPS wave 3 data collection was a fully sequential mixed mode design. The respondents were first offered to respond online by web mode (CAWI). CATI was offered at a later stage of data collection to sample members who had not responded to the web invitation. Next, CAPI was offered to those respondents who had not participated by Web or CATI. In the end, about 55% of the data was collected by web, 27% by telephone, and 18% by face-to-face interviews. The CAPI interviews employed show cards for some of the questions. The final response to the third wave of data collection was 4390 respondents. Since we are mainly interested in the mode effects in the third wave, we analyze the data of respondents who have responded to the third wave and also to the previous waves, leaving out nonrespondents on wave 2 or wave 3.

### Analysis methods

From the multi-item measures, we have selected 14 multi-item instruments that are assumed to be scales that measure a single underlying characteristic. Measuring an underlying characteristic by a scale has been referred to as *reflective measurement* (Bollen and Lennox, 1991). Some multi-item sets are not expected to form a scale, they are mere inventories of events or experiences that are expected to have an effect on respondents without reflecting an underlying characteristic. Such indices are referred to as formative measurement (Bollen and Lennox, 1991). Supplementary Material lists the multi-item scales used in our analysis.

When items are grouped in a scale, a Confirmatory Factor Analysis (CFA) can be used to check if they are indeed unidimensional and measure a single underlying characteristic. Multigroup CFA (MCFA) is then applied to evaluate their measurement equivalence across groups (research question 1). If the groups differ on some covariates, which indicates a selection process, various forms of adjustment are available. The correction approach used most often is conditioning on covariates that are related to the selection process and the target variables of the survey. Vannieuwenhuyze and Loosveldt (2013) call this approach *calibration*. The covariates are incorporated in the model by regressing the observed indicators on the covariates with equality constraints on the regression coefficients in all analyses, but allowing different intercepts (thresholds) across indicators and groups in the configural model. This follows the ANCOVA model described by Muthén (2002).

By regressing the observed indicators on the covariates, we assume that in the mixed mode design there is a selection on the observed variables. If the selection is on the latent variable instead, application of the Pearson–Lawley selection formulas leads to the conclusion that latent selection leads to an invariant factor model (Lawley and Maxwell, 1963; Meredith, 1964), even if the selection process is unknown. If the selection is on the observed indicators, application of the Pearson–Lawley selection formulae leads to the conclusion that the model is not invariant (Lawley and Maxwell, 1963; Muthén, 1989). We model the selection on the observed variables because in a general survey mostly demographics are available, which are expected to have only a small relationship with the latent variables. In a longitudinal survey we have access to measures from earlier measurement occasions, but we view these at best as proxies for the latent variables at later measurement occasions. By regressing the observed variables on the covariates, we expect that the factor model will change, in the direction of stronger measurement invariance.

When scale indicators have fewer than five categories we employ the ordered categorical variable methodology (Finney and DiStefano, 2006) as implemented in Mplus 7.1 (Muthén and Muthén, 1998-2012). For the measurement equivalence analysis, the consequence is that for categorical items the location parameter is no longer the intercept but a set of thresholds, which for scalar equivalence must be constrained equal across groups. Supplementary Material indicates which scales have categorical indicators, and provides reliability estimates (coefficient alpha) and some descriptive statistics for the scales.

If we have a large number of potential covariates, the covariate adjustment becomes unwieldy and also results in a complex model that estimates many regression coefficients for the covariates. To reduce the complexity of the model, that is, the number of covariates, propensity score methods can be used. Propensity scores were introduced by Rosenbaum and Rubin (1983) as a method to equalize an experimental and a control group on a set of covariates. The propensity score for a specific subject is the conditional probability of being assigned to treatment vs. control, given a set of covariates X. It can be viewed as a balancing score; a function *f*(X) of the covariates, such that the conditional distribution of the set of covariates X given *f*(X) is the same in both groups. The propensity score is used as a substitute for the entire set of covariates, thus considerably reducing the complexity of the model. Controlling for propensity scores can be performed by using them as covariates in an analysis (i.e., regression adjustment), or weights can be constructed based on the inverse of the propensity score (i.e., weighting adjustment). In our case we use regression adjustment. For a general overview of propensity score methods see Guo and Fraser (2010), for a review of propensity scores in surveys see Lee (2006).

Propensity scores are usually based on socio-demographic variables. This raises the question whether these are sufficient; propensity score methods assume that the propensity model includes all relevant variables. In longitudinal surveys, such as the NKPS, researchers have access to much richer information, namely the scores of respondents on the same variables collected on previous measurement occasions. For this reason we construct two different propensity scores: one based only on the socio-demographic variables and one based on the scales under investigation, measured in the previous wave that uses one single mode (face-to-face). Constructing a propensity score on the basis of observed sum scores in the first wave of data collection treats the scale scores as proxies for the latent variable scores at the first measurement occasion, which represents a stronger correction method than correcting on demographic information. Since having two sets of weights in one multivariate analysis is a complicated issue, we prefer applying the propensity score correction via regression adjustment. The first propensity score, based on demographics, is applied to answer research question two: “to what extent does measurement equivalence improve if selection on demographic variables is controlled for.” The second propensity score, based on previously measured scale scores, is added to the covariate based on demographics, to answer the third research question: “to what extent does measurement equivalence improve if scale scores from the earlier single mode data collections are added to the control variables.”

## Results

The Results section consists of two subsections. The first describes the construction of the propensity scores and the second presents the results of the measurement equivalence analyses.

### Construction of the propensity scores

There are several methods to construct propensity scores, the most popular being logistic or probit regression (Guo and Fraser, 2010), which results in one optimal regression equation predicting group membership. The propensity scores are the regression based predicted probabilities of group membership, which can then be used as a single covariate or as a weighting variable. This works well in a two group context where an experimental and a control group must be balanced. In our case, there are three groups (the three modes CAPI, CATI, CAWI) and using multinomial logistic regression therefore produces always two regression equations, each contrasting one mode with the reference mode in the coding system. In order to establish if one optimal equation for each set of predictors may be sufficient to calculate a single propensity score, we decided to use discriminant analysis, as this has the potential to produce fewer relevant regression equations. In a discriminant analysis of three groups, a discriminant function is constructed, basically a regression function, that maximally discriminates between these three groups simultaneously. Next, a second discriminant function is constructed that maximally discriminates the three groups under the constraint that the second discriminant function is uncorrelated with the first discriminant function. Since the discriminant functions maximize discrimination between groups, successive discriminant functions decrease in importance, and it is usual to find fewer significant discriminant functions than there are degrees of freedom (the number of groups minus one). For a detailed description of discriminant analysis we refer to Tabachnick and Fidell (2013).

The first discriminant analysis used only the demographic variables gender, age, education, and urbanization, as measured in wave 1. Urbanization was not a significant predictor, and the final discriminant analysis is based on the demographic variables gender, age and education. The first discriminant function captures 93.7% variance of the demographic variables, and a high score on this function reflects high age, being female and having a lower education. The second discriminant function explains 6.3% variance, and reflects being female with a high education. The high age, female and lower educated respondents represented by the first discriminant function are overrepresented in the CATI and CAPI modes and underrepresented in the CAWI mode in wave 3; the canonical correlation between this discriminant function and survey mode is 0.29. The female respondents with high education represented by the second discriminant function are underrepresented in CAPI, which indicates that they prefer to respond by telephone or web. Since the second discriminant function covers only 6% of the variance of the demographic variables, and since the associated canonical correlation with survey mode is only 0.08, it was decided to use only the first discriminant function as propensity score to correct for demographic differences. This propensity score is labeled D1 in the text and tables.

The second discriminant function is based on the scale scores in the second wave for those respondents who were interviewed using face-to-face. To avoid an accumulation of missing values in the scale scores, when some items of a scale were missing, scales were assigned the mean value on the available items. This was done after appropriate recoding for negatively worded questions and only if not more than 30% of the items of a scale were missing. If more items were missing, the scale score was assigned a missing value. A more serious missing value problem posed scales that apply only to a subsample of the respondents. For example, some scales enquire after parenting behavior, which of course only apply to respondents in certain age groups who actually have children. For all other respondents, such scales are assigned a missing value. Since SPSS Discriminant analysis uses listwise deletion to deal with missing values, simply specifying all available scales as potential predictors in a discriminant analysis would result in selecting only the small subgroup to which all of the scales apply. This not only dramatically reduces the number of respondents available for the analysis, but also restricts the analysis to a very specific subgroup of respondents. To avoid this, the discriminant analysis was carried out in a stepwise fashion. The first step includes as potential predictors all scales that apply to the entire sample, using forward selection to select only significant predictors. In the next steps, scales about partners and children were added. The scales about partners proved to be significant but the scales about children were not. Finally, a discriminant analysis was performed using all significant predictors. For the scales on partners, the missing values were imputed by the overall mean of the available values, and a dummy variable was added to indicate those cases where such imputation had taken place. As a result, the respondents to which these scales do not apply were not dropped from the analysis. The results of this discriminant analysis are summarized in Table 1.

**Table 1:** **Standardized canonical discriminant function coefficients**.

**Scale**	**Function**	
	**1**	**2**
Parental obligations	0.39	0.35
Parenthood	0.21	0.33
Loneliness	−0.20	−0.57
Conflicts partner	0.21	−0.00
Conflicts partner_missing	−0.79	0.43
Division homemaking tasks	0.30	−0.37
Division homemaking tasks_missing	0.07	0.08

The first discriminant function captures 89.1% variance of the wave 2 scales. A high score on this function reflects having a partner, feeling parental obligations, and division of homemaking tasks. The canonical correlation of this discriminant function with survey mode is 0.22. The second discriminant function explains 10.9% variance, and reflects having no partner combined with a low score on loneliness. Since the second discriminant function explains only 11% of the variance in the scales and the canonical correlation with survey mode is only 0.08, only the first discriminant function is used as propensity score. This propensity score is labeled D2 in the text and tables.

Summarizing: the first propensity score D1 reflects differences in the sample composition of the three modes in demographic characteristics, and the second propensity score D2 reflects differences between the three modes in their scale scores on the previous, single mode, measurement occasion.

### Measurement equivalence tests

To simplify interpretation of the equivalence tests, the discriminant scores were standardized. The propensity scores were included in the measurement model by treating them as observed covariates; that is, regressing all observed indicators on the propensity scores, with equality constraints on the regression coefficients across the three modes (Muthén, 2002). Partial measurement models were investigated only if full equivalence did not hold and if the modification indices suggested that a partial equivalence model could improve the model fit. In Table 2, the qualification of the measurement equivalence includes partial equivalence. Decisions on model fit were done using the chi-square difference test (Jöreskog, 1971) because the models tested against each other are nested. In the case of categorical variables (<5 categories) the adjusted chi-square was applied using the DIFFTEST option in Mplus.

**Table 2:** **Summary of results equivalence testing; (p) indicates partial equivalence**.

**Scale items (cat.) is categorical**	**Scale**	**No correction**	**Correction for D1**	**Correction for D1+D2**
8A – 8E (cat.)	Support partner	Scalar (p)	Scalar (p)	–^a^
9A – 9E (cat.)	Conflicts partner	Scalar (p)	Scalar (p)	Scalar (p)
10A – 10D	Quality partner relationship	Scalar (p)	Scalar (p)	Scalar (p)
11A – 11E	Division homemaking tasks	Configural	Configural	Configural
13A – 13D (cat.)	Activities with children	No scale^b^	Configural	Configural
14A – 14D	Division childrearing tasks	Metric	Scalar	Scalar
24A – 24D	Family responsibility expectations	Scalar (p)	–^a^	Scalar (p)
24E – 24H	Filial responsibility expectations	Configural	Configural	Configural
24I – 24L	Parental obligations	Configural	Configural	Scalar
24M – 24P	Parenthood	Scalar (different means)	Scalar (different means)	Scalar (different means)
30A – 30D (cat.)	State vs. family	No scale^b^	No scale^b^	No scale^b^
32A – 32E	MHI-5	Metric	Configural	Metric
33A – 33K (cat.)	Loneliness	Metric (p)	Scalar (p)	Metric (p)
35M – 35P	Satisfaction with life	Scalar (p) (different means)	Scalar (p)	Metric (p)

*The χ2 difference test for categorical analyses is computed using DIFFTEST*.

a *“–” Indicates that after imposing full scalar equivalence, the model did not fit adequately, but modification indices did not point to specific improvements to the model*.

b *RMSEA>0.10 and CFI\TLI < 0.90*.

Table 2 indicates that full scalar measurement equivalence is rare for these scales. Correction for demographics (D1), or demographics plus wave two scales (D1+D2), in general improved the measurement equivalence. To explain the models behind the summaries in Table 2, we use (1) the Division Homemaking Tasks scale (scale 4), as example of a scale that has only configural equivalence; (2) the Parental Obligations scale (scale 9), as example of a scale where measurement equivalence clearly improves after propensity score correction; and (3) the Division of Childrearing Tasks scale (scale 6), as example of a scale that shows good measurement equivalence throughout.

### Configural equivalence: the division homemaking tasks scale

The Division Homemaking Tasks scale showed only configural invariance, meaning that the same factor structure can be imposed on these five items. The chi-square for the data with no correction is χ^2^_(15)_ = 170.9, *p* < 0.001, and values of the fit indices are RMSEA = 0.10 and CFI = 0.96. The model fit improved when corrections for selection effects were made. When we correct for demographics (D1) the chi-square is χ^2^_(23)_ = 183.0, *p* < 0.001 with RMSEA = 0.07 and CFI = 0.96. When both propensity scores (i.e., demographic D1 and previous wave scale scores D2) are used for correction, the chi-square for the configural equivalence model becomes χ^2^_(31)_ = 189.4, *p* < 0.001, with RMSEA = 0.06 and CFI = 0.96. Even with propensity score corrections, stronger levels of measurement equivalence than configural were not reached. Table 3 presents the factor loadings and error variances for all data collection modes for the final configural equivalence model including the D1+D2 propensity score correction.

**Table 3:** **Factor loadings and intercepts Division Homemaking Tasks after D1+D2 propensity score correction: Configural equivalence**.

**Item**	**Loadings**			**Intercepts**		
	**CAPI**	**CATI**	**CAWI**	**CAPI**	**CATI**	**CAWI**
11A	1.00	1.00	1.00	2.82	2.55	2.48
11B	0.66	0.65	0.78	2.83	2.68	2.58
11C	0.75	0.69	0.72	2.69	2.55	2.45
11D	−0.10	−0.05	−0.11	2.87	3.03	2.81
11E	−0.41	−0.47	−0.54	3.00	3.20	3.12

Although the data for this scale do not support either metric or scalar equivalence, it is clear that the loadings are nevertheless rather similar across the measurement modes. In fact, the correlation between any two columns of loadings is above 0.99. So it is tempting to invoke some kind of robustness and claim that modes can be combined and analyzed together, because the errors that are induced by this formally incorrect combination procedure are small and can be safely ignored. We come back to this in our discussion.

### Improvement with propensity score correction: the parental obligations scale

The Parental Obligations scale provides a nice example of improvement in measurement quality when the propensity score correction for selection is taken into account. Without adjustment, the chi-square for the configural equivalence model is χ^2^_(6)_ = 25.5, *p* < 0.001, and the fit indices are RMSEA = 0.05 and CFI = 1.00. Metric or scalar equivalence cannot be established. When we correct for demographics (D1) the chi-square for the configural equivalence model is χ^2^_(12)_ = 38.8, *p* < 0.001, and the fit indices are RMSEA = 0.04 and CFI = 1.00. Again, no metric or scalar equivalence can be established. With adjustment for both propensity scores (i.e., demographic D1 and previous wave scale scores D2) the chi-square for the strong scalar equivalence model becomes χ^2^_(32)_ = 69.2, *p* < 0.001 with fit indices RMSEA = 0.03 and CFI = 0.99. The fit indices are well within conventional limits for good fit, and we conclude that after D1+D2 correction full scalar equivalence is reached. To illustrate the effect of adding the correction for scale scores on the previous wave to the demographics, Table 4 shows the factor loadings for the three modes in the configural model after D1 correction and under the heading *All* the common loadings in the final full scalar equivalence model (after D1+D2 correction).

**Table 4:** **Factor loadings and intercepts error variances Parental Obligations Scale, for configural model after D1 correction and full scalar equivalence model after D1+D2 correction (All)**.

**Item**	**Loadings**				**Intercepts**			
	**CAPI**	**CATI**	**CAWI**	**All**	**CAPI**	**CATI**	**CAWI**	**All**
24I	1.00	1.00	1.00	1.00	2.41	2.48	2.53	2.49
24J	1.26	1.15	1.19	1.19	2.95	3.01	3.08	3.03
24K	1.02	1.02	1.18	1.14	2.66	2.69	2.81	2.74
24L	0.72	0.67	0.78	0.71	3.40	3.40	3.50	3.45

In this example it is clear that using propensity score adjustment based on both demographics and previous wave scale scores leads to full scalar equivalence, which allows analyzing all data disregarding mode effects. It is interesting that without correction for the D2 propensity scores this is not the case. Again, we could argue that the loadings are very similar across the three modes, but in this case it is obviously better to use SEM analysis for the substantive research questions, including the two propensity scores as covariates in all analyses.

### Full metric and scalar equivalence throughout: the division of childrearing tasks scale

The Division of Childrearing Tasks scale shows full metric equivalence without correction, and reaches full scalar equivalence with either correction for only D1 (i.e., demographics) and for correction for both D1 and D2 (i.e., demographic plus previous wave scale) propensity scores. Without adjustment the chi-square for the metric equivalence model is χ^2^_(12)_ = 15.2, *p* = 0.23, and the fit indices are RMSEA = 0.02 and CFI = 1.00. After correction for demographics (D1) we have a scalar equivalence model with χ^2^_(26)_ = 36.5, *p* = 0.08, and the fit indices are RMSEA = 0.02 and CFI = 0.99. After correction for both demographics (D1) and previous wave scale scores (D2) this marginally improves into a scalar equivalence model with χ^2^_(32)_ = 41.3, *p* = 0.13, and the fit indices are RMSEA = 0.01 and CFI = 1.00.

Table 5 shows the loadings and intercepts of the models without correction (metric equivalence) and with correction for D1+D2 (full scalar equivalence). It is clear that adding covariates to the model brings the intercepts closer together, but from one model to the next the changes are very small.

**Table 5:** **Loadings and intercepts division childrearing tasks scale**.

**Item**	**Uncorrected item scores**				**Correction D1**		**Correction D1 + D2**	
	**Loadings**	**Intercepts**			**Loadings**	**Intercepts**	**Loadings**	**Intercepts**
		**CAPI**	**CATI**	**CAWI**				
14A	1.00	2.57	2.66	2.76	1.00	2.39	1.00	2.32
14B	1.21	2.63	2.48	2.67	1.27	2.45	1.27	2.34
14C	0.63	2.79	2.72	2.79	0.66	2.68	0.66	2.63
14D	1.10	2.74	2.56	2.70	1.13	2.48	1.11	2.37

## Conclusion and discussion

In this study, we addressed three related research questions. The first question is if the examined NKPS scales show measurement equivalence. The answer is that by and large they do, but in most cases we reach only partial measurement equivalence. The second research question is to what extent measurement equivalence improves if selection on demographic variables is controlled, and the third research question is to what extent measurement equivalence improves if scale scores from earlier, single mode, data collections are added to the control variables. In general, our analyses show that measurement equivalence improves if selection is controlled for, and that these measurement improvements improve more if in addition to demographics also previous wave scale scores are controlled for. Apparently, besides standard demographics, responses on an earlier wave play a role too. However, controlling for selection is not a panacea; there are a few cases where it does not improve the measurement equivalence at all, and one case (i.e., scale with items on activities with children) were adding the previous scale scores as covariate actually produces a weaker level of measurement equivalence.

One reviewer raised the question why correcting for propensity scores, which are a summary of demographic differences and scale score differences on the previous wave, only improves measurement equivalence in four out of 14 scales. One reason is that propensity score adjustment aims to correct for differential selection of respondents into specific modes. In addition to selection, our results point toward real mode effects in the measurement process. Berzelak (2014) makes a very useful distinction between mode inherent factors and context specific and implementation specific characteristics (see also De Leeuw and Berzelak, 2014). Mode inherent factors are given; examples are the involvement of interviewers in face-to-face and telephone surveys, absence of visual design elements in aural survey modes. Such factors are always present in specific modes. Context specific characteristics depend on social and cultural factors, such as familiarity with technology in the target population. These characteristics are difficult to influence, although they are likely to change over time. Implementation specific characteristics depend on the way a specific mode is actually implemented, such as the use of specific visual design elements in paper and web surveys. These are in principle under control of the researchers, and may be managed in a way to counteract context specific or mode inherent factors. The relatively small impact of our adjustment on the level of measurement equivalence suggests that mode inherent and context and implementation factors may be more important in mixed mode surveys than differential selection processes. If this is the case, research into mode effects and adjustment methods should attempt to include these characteristics, for example by collecting and using more paradata (Kreuter, 2015).

The results that we find depend of course on particularities of the instruments, data collection procedures, and sampling design employed in the NKPS. As large scale studies tend to make the switch from the expensive face-to-face mode to other modes, including mixed mode designs, other data will become available to investigate the generalizability of our results. In addition, it would be informative to carry out simulation research that manipulates potential selection mechanisms and employs different correction strategies.

The ideal situation is, of course, full scalar equivalence across modes. If full scalar equivalence is reached, we are justified in using scale sum scores in our analysis. If partial scalar equivalence is reached, such sum scores can be misleading, but scale means can be compared in structural equation models that include a partially equivalent measurement model. When only metric equivalence is reached, statements about differences in means, whether observed sum scores or factor means in a structural equation model, are not supported and cannot be validly made, but statements about covariances and correlations are still valid. When merely configural equivalence is reached, even statements about correlations can be invalid. In our analysis of the 14 NKPS scales, we find seven instances of (partial) scalar equivalence and three instances of (partial) metric equivalence. In three instances we find configural equivalence, and in one instance (state versus family support) the analysis shows that the items are not forming a scale according to any reasonable criterion.

If configural equivalence is established, we are measuring the same construct, but we measure it in slightly different ways in the different modes. If the actual values of the intercepts and loadings are close to each other across survey modes, as is the case in our example of the Division of Homemaking Tasks scale, it becomes very tempting to argue for some kind of robustness, even when metric or partly scalar equivalence does not hold. If the intercepts and loadings are very close, analysts might make a leap of faith, simply ignore any differences in intercepts and loadings, and work with SEM analyses of the combined data set or even compute sum scores for the scales and work with these, again on the entire data set. In our view, this may be defensible from a practical standpoint, but the burden of proof is on the researchers. They should make an attempt to estimate the amount of distortion produced by ignoring the real differences between intercepts and loadings across modes and demonstrate that the substantive effects they want to interpret are clearly larger than these measurement differences. Since analyses that follow this approach work by sweeping some real but hopefully small differences under the carpet, robust standard errors or bootstrapping should always be used to assess the real uncertainty in this case, since asymptotic statistical methods will underestimate the sampling variance.

A different way to deal with small measurement differences between survey modes is to employ a model that allows them and includes them explicitly in the model. Bayesian estimation is actually able to accomplish this, by introducing difference parameters in the model and by posing a prior distribution with a small variance for the difference parameters. For an example we refer to van de Schoot et al. (2013). This is a new and promising approach, but this is also an area that in our view needs more simulations and robustness studies to investigate when this approach works well and when it does not. We recommend that analysts that follow this approach carry out a sensitivity analysis to demonstrate that the specific choice of a prior does not have a large effect on the results for the substantive research questions.

There is a different approach to lack of measurement equivalence, which we have not explored in this study, because in our data the number of items in a scale was rather small (4-5). If there are enough items to form a scale there is always the option of dropping an item to improve the scale properties. The bare minimum to have a testable measurement model is four items for each latent variable and the bare minimum for testing measurement equivalence is three items (cf. Hair et al., 2010). Hence, if the number of items is larger than three or four, there is the option of finding the item that shows the largest amount of measurement non-equivalence and removing that particular item from the analysis. It follows that if the study is in a phase of developing measurement instruments and a mixed mode data collection is considered, it makes perfect sense to design measurement instruments with more than four or five items. From a SEM measurement point of view, this produces a number of potential superfluous items, that can in the analysis stage be sacrificed on the altar of measurement equivalence, and still leave a measurement model large enough that it can be tested.

A limitation in our discussion is that we have addressed mainly the issues that arise after the mixed mode data collection has been carried out. There is a large literature on designing questionnaires and fieldwork procedures that are aimed at minimizing mode effects by careful design. This is a broad topic, which is beyond the scope of this paper; for an extensive review of the issues that arise in designing mixed mode surveys we refer to De Leeuw et al. (2008) and Dillman et al. (2014).

Finally, we note that to distinguish between selection and mode measurement effects we need auxiliary information. In our analyses we used demographic data and data from a previous single-mode measurement occasion. Often the assumption is made that questions on factual demographic data are insensitive to mode measurement effects; in our case this information came from register data available from Statistics. Netherlands. Auxiliary information is also needed when attempts are made to adjust for mode measurement effects. Vannieuwenhuyze et al. (2011) discuss methods that use auxiliary data from a single-mode reference survey. Klausch et al. (in preparation) present a framework that uses a repeated single-mode survey on the same respondents, a design that in fact applies to panel surveys such as the NKPS where at least one measurement occasion is single-mode. De Leeuw (2005) and De Leeuw and Hox (2011) suggest to embed r real experimental design in the mixed-mode survey by assigning a subset of respondents at random to survey modes instead of allowing self-selection. All these approaches provide information needed to disentangle selection and measurement effect, which is a prerequisite to adjustment. Again, adjustment is a broad topic, and beyond the scope of this paper. However, it is important that when survey researchers design a mixed mode study, they anticipate the possible emergence of selection and measurement effects, and they must design the data collection in such a way that the necessary auxiliary information is made available.

### Conflict of interest statement

The reviewer Jelte Wicherts declares that, despite being affiliated at the same department as the author Eva Zijlmans, the review process was handled objectively and no conflict of interest exists. The authors declare that the research was conducted in the absence of any commercial or financial relationships that could be construed as a potential conflict of interest.
