# Supplementary material for: Measurement equivalence in mixed mode surveys
Source: Front Psychol. 2015 Feb 5;6:87. doi: 10.3389/fpsyg.2015.00087 (PMC4318282; doi:10.3389/fpsyg.2015.00087)
Supplement: Supplementary file 1 [file DataSheet1.DOCX]

| Supplementary Material: Descriptions of scales used in the analysis. | | | | | | |
| --- | --- | --- | --- | --- | --- | --- |
| Items | Scale | Variable names | Number of categories | Cronbach’s Alpha | Mean | Standard deviation |
|  |  |  |  |  |  |  |
| 8A – 8E (categorical) | Support partner | as8a-as8e | 4 | 0.92 | 3.34 | 0.56 |
|  |  | bs8a-bs8e | 4 | 0.93 | 3.37 | 0.55 |
|  |  | cs8a-cs8e | 4 | 0.91 | 3.29 | 0.56 |
| 9A – 9E (categorical) | Conflicts partner | as9a-as9e | 3 | 0.90 | 1.33 | 0.32 |
|  |  | bs9a-bs9e | 3 | 0.90 | 1.35 | 0.33 |
|  |  | cs9a-cs9e | 3 | 0.89 | 1.29 | 0.30 |
| 10A – 10D | Quality partner relationship | as10a-as10d | 5 | 0.95 | 1.56 | 0.71 |
|  |  | bs10a-bs10d | 5 | 0.95 | 1.59 | 0.73 |
|  |  | cs10a-cs10d | 5 | 0.95 | 1.65 | 0.68 |
| 11A – 11E | Division homemaking tasks | as11a-as11e | 5 | 0.69 | 2.90 | 0.86 |
|  |  | bs11a-bs11e | 5 | 0.69 | 2.92 | 0.85 |
|  |  | cs11a-cs11e | 5 | 0.68 | 2.90 | 0.84 |
| 13A – 13D (categorical) | Activities with children | as13a-as13d | 4 | 0.61 | 1.84 | 0.57 |
|  |  | bs13a-bs13d | 4 | 0.98 | 1.88 | 0.59 |
|  |  | cs13a-cs13d | 4 | 0.86 | 1.78 | 0.60 |
| 14A – 14D | Division childrearing tasks | as14a-as14d | 5 | 0.83 | 2.69 | 0.80 |
|  |  | bs14a-bs14d | 5 | 0.82 | 2.70 | 0.76 |
|  |  | cs14a-cs14d | 5 | 0.79 | 2.69 | 0.73 |
| 24A – 24D | Family responsibility expectations | as24a-as24d | 5 | 0.87 | 2.29 | 0.75 |
|  |  | bs24a-bs24d | 5 | 0.87 | 2.36 | 0.70 |
|  |  | cs24a-cs24d | 5 | 0.86 | 2.42 | 0.73 |
| 24E – 24H | Filial responsibility expectations | as24e-as24h | 5 | 0.75 | 3.11 | 0.78 |
|  |  | bs24e-bs24h | 5 | 0.76 | 3.17 | 0.73 |
|  |  | cs24e-cs24h | 5 | 0.73 | 3.33 | 0.70 |
| 24I – 24L | Parental obligations | as24i-as24l | 5 | 0.78 | 2.80 | 0.76 |
|  |  | bs24i-bs24l | 5 | 0.79 | 2.89 | 0.74 |
|  |  | cs24i-cs24l | 5 | 0.78 | 2.94 | 0.72 |
| 24M – 24P | Parenthood | as24m-as24p | 5 | 0.81 | 4.02 | 0.80 |
|  |  | bs24m-bs24p | 5 | 0.83 | 4.06 | 0.78 |
|  |  | cs24m-cs24p | 5 | 0.81 | 4.06 | 0.70 |
| 30A – 30D (categorical) | State versus family | as30a-as30d | 4 | 0.83 | 2.40 | 0.59 |
|  |  | bs30a-bs30d | 4 | 0.82 | 2.36 | 0.57 |
|  |  | cs30a-cs30d | 4 | 0.81 | 2.37 | 0.57 |
| 32A – 32E | MHI-5 | as32a-as32e | 6 | 0.86 | 4.75 | 0.78 |
|  |  | bs32a-bs32e | 6 | 0.85 | 4.80 | 0.74 |
|  |  | cs32a-cs32e | 6 | 0.83 | 4.90 | 0.73 |
| 33A – 33K (categorical) | Loneliness | as33a-as33k | 3 | 0.86 | 1.27 | 0.27 |
|  |  | bs33a-bs33k | 3 | 0.94 | 1.28 | 0.28 |
|  |  | cs33a-cs33k | 3 | 0.93 | 1.22 | 0.25 |
| 35M – 35P | Satisfaction with life | as35m-as35p | 5 | 0.83 | 2.33 | 0.71 |
|  |  | bs35m-bs35p | 5 | 0.84 | 2.31 | 0.69 |
|  |  | cs35m-cs35p | 5 | 0.81 | 2.18 | 0.65 |
| Note: variables labeled *a* are wave 1, *b* are wave 2, and *c* are wave 3 variables. The mean and standard deviation refer to the scale score expressed as the mean of the items. | | | | | | |
